# Deep Learning Model for Predicting Intradialytic Hypotension Without Privacy Infringement: A Retrospective Two-Center Study

**DOI:** 10.3389/fmed.2022.878858

**Published:** 2022-07-07

**Authors:** Hyung Woo Kim, Seok-Jae Heo, Minseok Kim, Jakyung Lee, Keun Hyung Park, Gongmyung Lee, Song In Baeg, Young Eun Kwon, Hye Min Choi, Dong-Jin Oh, Chung-Mo Nam, Beom Seok Kim

**Affiliations:** ^1^Department of Internal Medicine, Yonsei University College of Medicine, Seoul, South Korea; ^2^Department of Biostatistics and Computing, Yonsei University Graduate School, Seoul, South Korea; ^3^Department of Internal Medicine, Myongji Hospital, Hanyang University College of Medicine, Goyang, South Korea; ^4^Division of Biostatistics, Department of Biomedical Systems Informatics, Yonsei University College of Medicine, Seoul, South Korea

**Keywords:** deep learning, intradialytic hypotension, machine learning, privacy protection, hemodialysis

## Abstract

**Objective:**

Previously developed Intradialytic hypotension (IDH) prediction models utilize clinical variables with potential privacy protection issues. We developed an IDH prediction model using minimal variables, without the risk of privacy infringement.

**Methods:**

Unidentifiable data from 63,640 hemodialysis sessions (26,746 of 79 patients for internal validation, 36,894 of 255 patients for external validation) from two Korean hospital hemodialysis databases were finally analyzed, using three IDH definitions: (1) systolic blood pressure (SBP) nadir <90 mmHg (Nadir90); (2) SBP decrease ≥20 mmHg from baseline (Fall20); and (3) SBP decrease ≥20 mmHg and/or mean arterial pressure decrease ≥10 mmHg (Fall20/MAP10). The developed models use 30 min information to predict an IDH event in the following 10 min window. Area under the receiver operating characteristic curves (AUROCs) and precision-recall curves were used to compare machine learning and deep learning models by logistic regression, XGBoost, and convolutional neural networks.

**Results:**

Among 344,714 segments, 9,154 (2.7%), 134,988 (39.2%), and 149,674 (43.4%) IDH events occurred according to three different IDH definitions (Nadir90, Fall20, and Fall20/MAP10, respectively). Compared with models including logistic regression, random forest, and XGBoost, the deep learning model achieved the best performance in predicting IDH (AUROCs: Nadir90, 0.905; Fall20, 0.864; Fall20/MAP10, 0.863) only using measurements from hemodialysis machine during dialysis session.

**Conclusions:**

The deep learning model performed well only using monitoring measurement of hemodialysis machine in predicting IDH without any personal information that could risk privacy infringement.

## Introduction

Intradialytic hypotension (IDH) is one of the most frequent complications in patients requiring maintenance hemodialysis. IDH is associated with an increased risk of cardiovascular and all-cause mortality (1–4). The definition of IDH varies among studies, while the prevalence of IDH ranges up to 40% (5). Although the risk factors involved in IDH are well known, including diabetes, cardiovascular disease, autonomic dysfunction, nutrition status, old age, anemia, and high interdialytic weight gain, most of these risk factors are difficult to correct immediately at the hemodialysis center. Therefore, treatments, such as temporarily stopping hemodialysis or reducing the rate of ultrafiltration, are preferentially performed when IDH occurs. In order to detect IDH early, it may be helpful to measure blood pressure (BP) more frequently, however, it is impossible to measure BP continuously due to the nature of the non-invasive BP measurement method. Thus, other non-invasive methods that can predict IDH in advance are needed.

However, it is difficult to predict IDH with a classical statistical model, because there many different factors play a role in IDH. Recently, some studies have reported prediction of IDH in hemodialysis patients by using machine learning or deep learning, approaches that can process multi-dimensional data (6–8). However, these models require various types of clinical information, some of which pose a risk of a breach of privacy. Additionally, because each prediction model developed in these studies used different variables, the models could not be applied in all hemodialysis units for universal use. On the other hand, various measurements, including blood flow rate, ultrafiltration rate, dialysate flow rates, arterial line pressure, venous line pressure, transmembrane pressure, temperature, bicarbonate level, and sodium level, can be generated during hemodialysis sessions. These values can be measured by any hemodialysis machine, from different manufacturers, can be used as variables in all hemodialysis units, and do not pose a high risk of privacy infringement.

Therefore, in this two-center validation study, we aimed to develop a real-time IDH prediction model using only data generated from the hemodialysis machine, excluding any personal information, to validate the performance of the developed models.

## Materials and Methods

### Study Population

**Figure 1:**
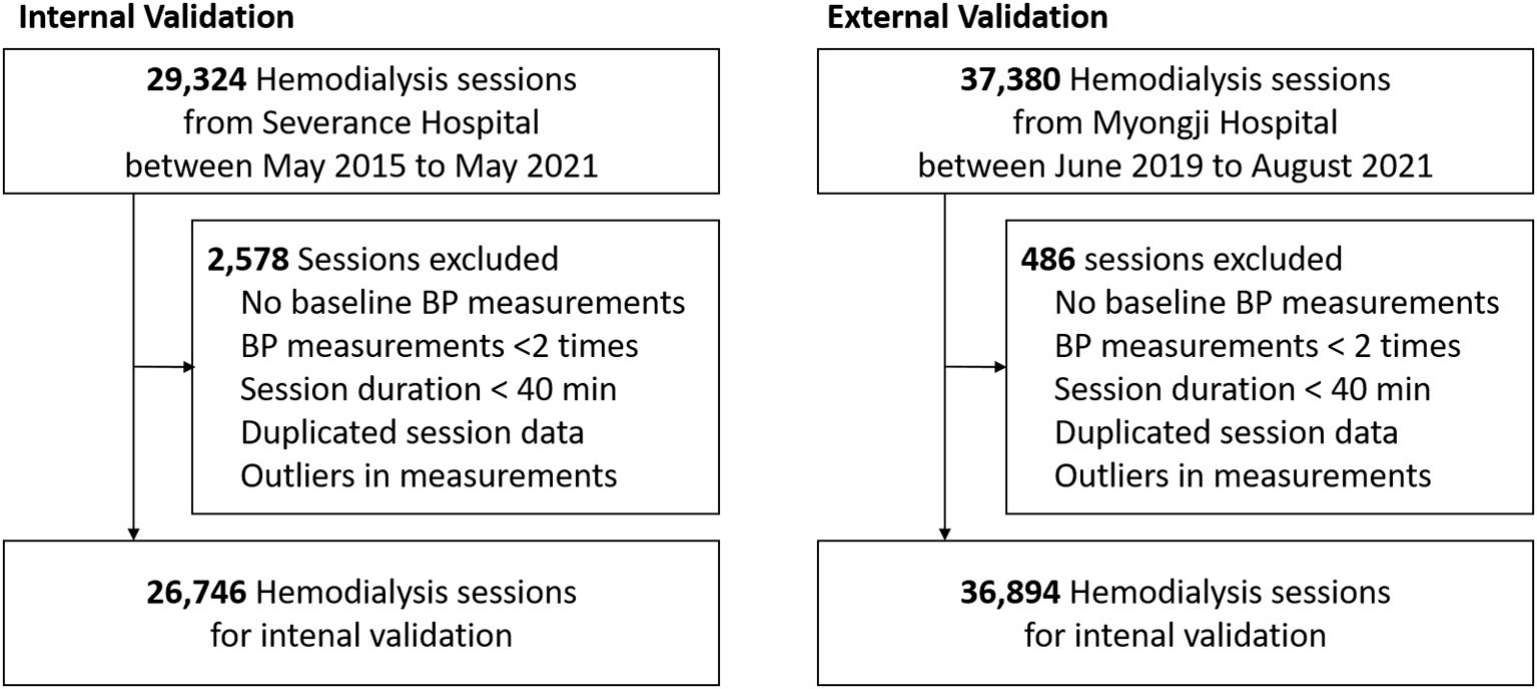
Flowchart of this study. BP, blood pressure.

Hemodialysis data were extracted from the databases of two hospitals in Korea (Severance Hospital and Myongji Hospital), which store information about each hemodialysis session. In total, 29,324 sessions of 135 patients aged over 19 years, which were automatically recorded in the Therapy Data Management System from May 2015 to May 2021, in Severance Hospital, and 37,380 sessions of 255 patients aged over 19 years, which were automatically recorded in the same system from June 2019 to August 2021, in Myongji Hospital, were screened. Among these 66,704 sessions, we excluded the following sessions: (1) sessions without consecutive BP measurements; (2) sessions without ultrafiltration; (3) sessions lasting less than 40 min; (4) no baseline BP measurement obtained within first 10 min; (5) sessions with missing variables; (6) sessions considered as containing other input errors (Figure 1).

Finally, 63,640 sessions involving 387 patients were included in this study. The study was performed following the Declaration of Helsinki principles. The institutional review boards of Severance Hospital (IRB No. 4-2021-0951) and Myongji Hospital (2021-08-022) approved this study and waived the need for obtaining informed patient consent, as only de-identified, previously collected data were accessed.

### Hemodialysis Sessions

Each hemodialysis session was automatically saved to the database. Along with the initial hemodialysis settings, measured values, including arterial line pressure (AP), venous line pressure (VP), blood flow rate, dialysate flow rate, ultrafiltration rate, total ultrafiltration volume, temperature, and dialysate sodium level were collected from the hemodialysis machine every minute. Vital signs, including systolic blood pressure (SBP), diastolic blood pressure (DBP), mean arterial pressure (MAP), and pulse rate, were recorded every 1 h by default, and all additional measurements were also recorded at any time-point during hemodialysis session. Blood pressure was additionally measured when patient complained of any symptoms associated abnormal blood pressure.

### Outcomes and Additional Variables

IDH was defined as a nadir SBP < 90 mmHg (Nadir90) or as a decrease in SBP ≥ 20 mmHg compared to the initial baseline BP (Fall20) within 10 min. In addition, it was also defined as a decrease in systolic BP ≥ 20 mmHg and/or a decrease in MAP of ≥ 10 mmHg (Fall20/MAP10) within 10 min. Age and sex were the only demographics collected from sessions. No other clinical information that could infringe privacy was collected.

### Data Processing

**Figure 2:**
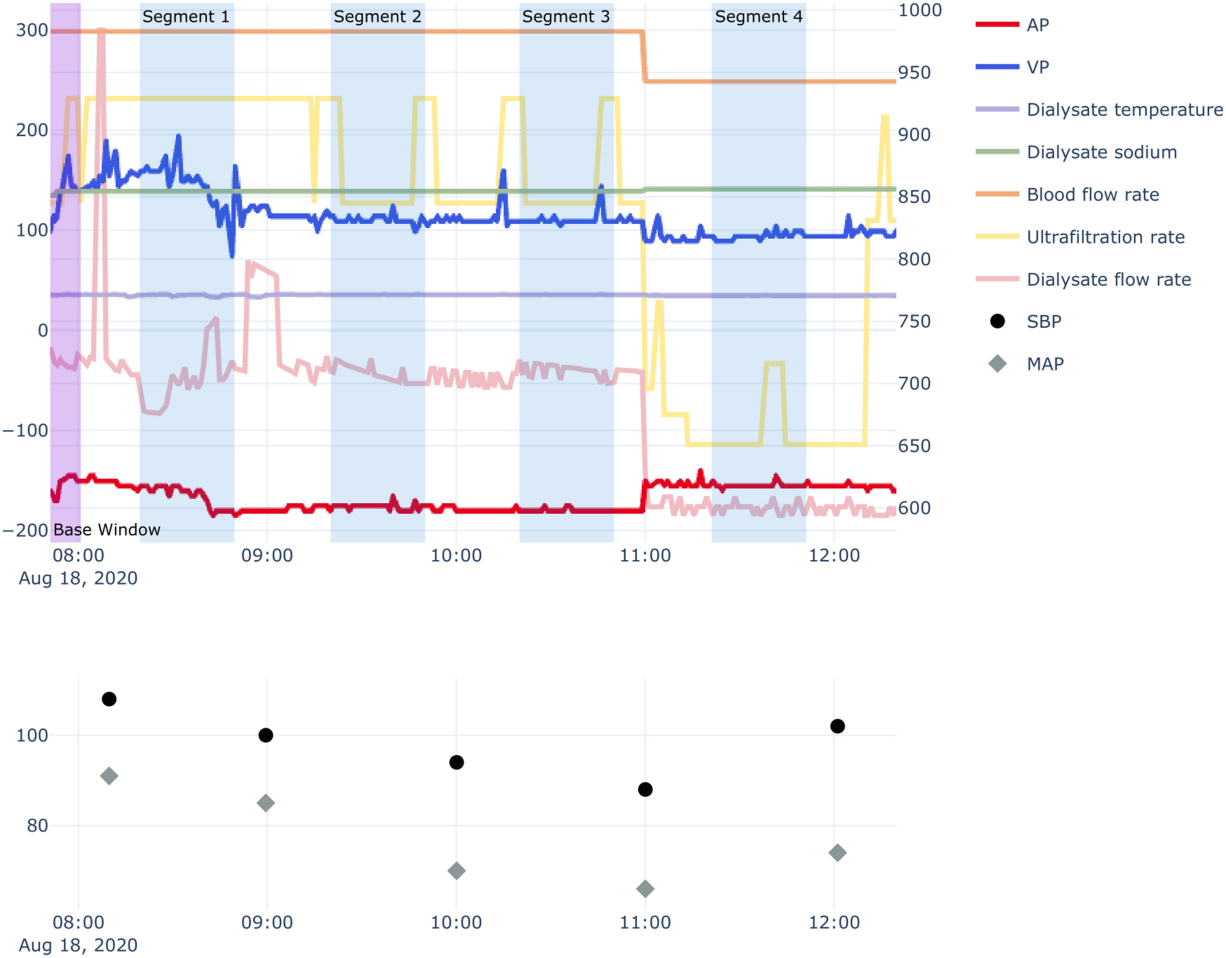
Example of hemodialysis session and segmentation recognition. AP, arterial pressure; VP, venous pressure; SBP, systolic blood pressure; MAP, mean arterial pressure. Segments contain hemodialysis-related measurement data obtained for 30 min, 10 min before each time-point of SBP measurement. Bottom panel: 10 min of data from the start of hemodialysis were used to adjust the volatility of the AP and VP for each session.

In this study, to predict IDH within 10 min, a series of hemodialysis-related measurements obtained during the previous 30 min were collected in real time. Therefore, each segment was defined as 30 min from 40 min to 10 min before any time point at which SBP was measured (Figure 2). A segment had 30 time-points because variables from hemodialysis machines were measured every minute. We used 10 min of data from the start of hemodialysis as baseline data to adjust the volatility of AP and VP for each session and calculated the mean values of the AP and VP from baseline data for each session. Then, the calculated mean values were subtracted from the original AP and VP values.

In addition, we created variables to obtain better performance for predicting IDH. To consider the possibility that the passage of time may influence the occurrence of IDH events, we used two additional variables related to the passage of time in each hemodialysis session: (1) the time elapsed from the initiation of the hemodialysis session to the beginning of each segment; (2) the time elapsed from the previous SBP measurement to the beginning of each segment. For analyses using logistic regression, random forest (9), and Extreme Gradient Boosting (XGBoost) methods (10), we extracted the mean and standard deviation (SD) of time-varying variables for each segment.

### Model Development and Validation

We developed a deep learning model to predict IDH using a convolutional neural network (CNN). CNNs have generally been used to analyze image data, such as classification, segmentation, and object detection (11–14). They can also be used to detect abnormal events in time-series data (15–18). We processed 10 time-invariant variables as a one-dimension (1-D) array with a fixed value. Then, the time-varying and time-invariant variables were concatenated as a 1-D array with 30 time-points and channels corresponding to the number of variables. Then, we can extract new features that considers the relationship between various variables, including the relationship between time-invariant and time-variant variables through 1-D convolutional layer.

**Figure 3:**
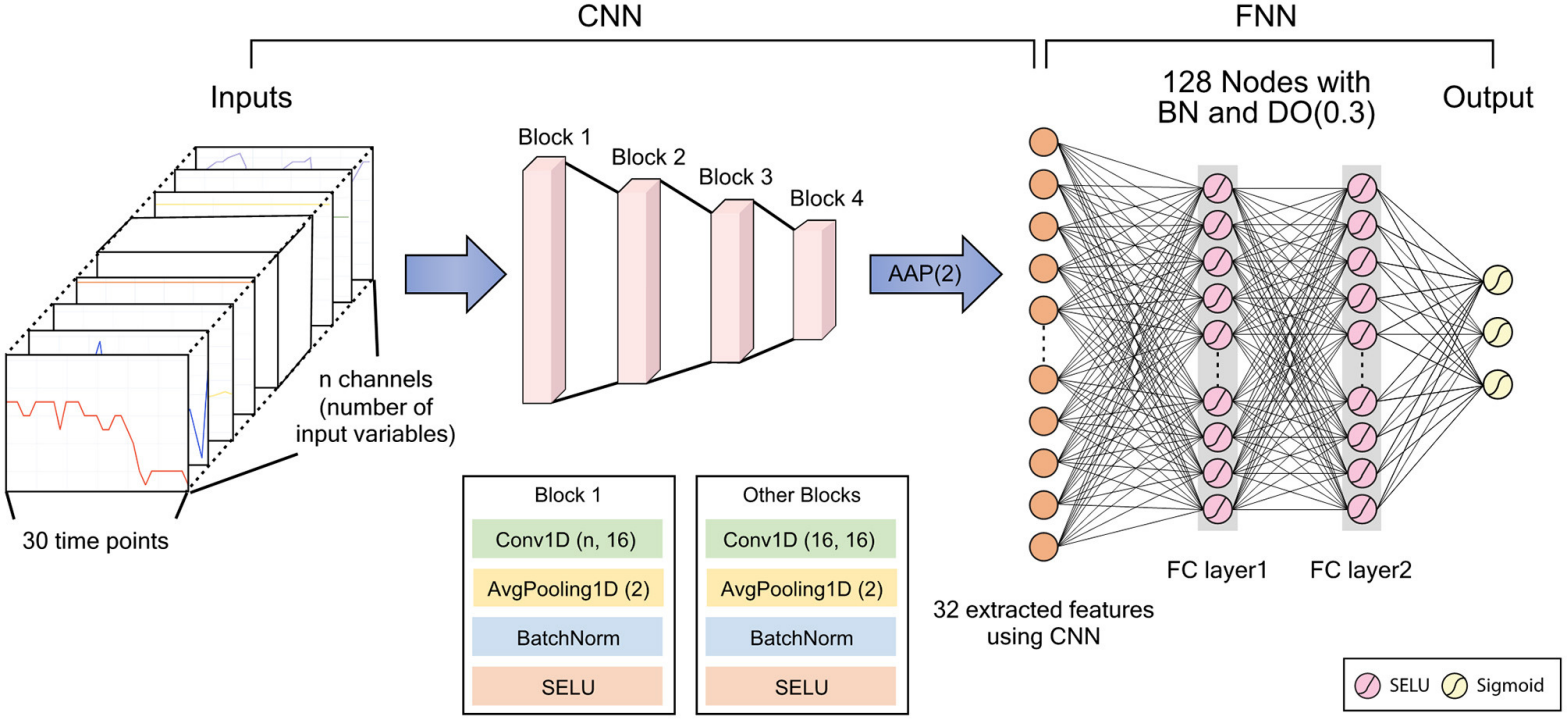
Architecture of the deep learning model. CNN, convolutional neural network; FNN, feedforward neural network; BN, batch normalization; DO, dropout; AAP, adaptive average pooling; SELU, scaled exponential linear unit; FC, fully connected. DO (dropout rate), Conv1D (kernel size, number of filters), AvgPooling1D (kernel size).

Our deep learning model used several features extracted through the CNN, which consisted of four blocks and one 1-D adaptive average pooling layer in a row. The block was composed of one 1-D convolutional layer, one batch normalization, one Scaled Exponential Linear Unit (SELU) activation function, and one 1-D average pooling layer in order. Then, the concatenated 1D layer was connected to an output layer via two fully connected layers, with 128 nodes, three SELU activation functions, and one dropout layer, with a 0.3 dropout rate. Since three IDH events could occur at the same time, we set output layer to return three predicted probabilities of IDH through the sigmoid activation function. Therefore, our deep learning model estimates probabilities for three different IDH events simultaneously. The detailed architecture of our deep learning model is illustrated in Figure 3.

To train the deep learning model, we used the Adam optimizer (19), binary cross entropy loss, and a batch size of 64. We also used the one-cycle scheduler to reduce training time (20). In the random forest model, nodes were expanded until all leaves are pure or until all leaves contain less than the minimum number of samples required to split, and the Gini index was used to measurement of the quality of a split. The maximum tree depth in XGBoost model was set to 6, and the number of boosting rounds was set to 100. Detailed hyperparameter settings for the random forest and XGBoost model are summarized Supplementary Table S1. We trained the machine learning and deep learning models using the data from the Severance Hospital hemodialysis database only (internal validation) and used the data from the Myongji Hospital hemodialysis database for external validation. The internal validation was performed through the 5-fold cross-validation.

### Model Interpretation

We used the SHapley Additive exPlanation (SHAP) analysis to assess variable importance and the effect of variables on IDH for our deep learning model (21). The SHAP value is based on the Shapley value, which is a solution concept in cooperative game theory. The SHAP value can be interpreted as how much a certain value of a variable affects the predicted value. Unlike standard variable importance, SHAP values are calculated for the subject-specific contributions of each variable. Therefore, it is possible to determine the effects of variables that may differ from subject to subject, as well as overall impact of variable. In this study, we accessed the effect of variables on IDH prediction over time as well as the overall variable importance.

### Statistical Analyses

We used descriptive statistics to describe covariates. Categorical variables are expressed as number of patients with percentage, while continuous variables are presented as mean with SD. Standard deviation was previously defined in the Data processing section. For performance evaluation, we use the AUROC and the area under the precision-recall curve (AUPRC) as performance measurements. We calculated these performance measures through 5-fold cross-validation. We performed a sensitivity analysis to determine which variables had the most impact on IDH prediction. We divided variables into several groups: monitored pressure (AP, VP), setting measures (blood flow rate, dialysate flow rate, ultrafiltration rate, total ultrafiltration volume, temperature, and sodium), vital signs (SBP, DBP, MAP, and pulse rate), and time information. We trained models by excluding each group and evaluated these trained models. In addition, we also included demographic variables, such as age and sex, in building the models, to assess whether other clinical variables affected the performance of IDH prediction. Furthermore, since the ultrafiltration rate may have a different effect on the IDH events depending on the pre-dialysis weight, we conducted additional analysis using normalized ultrafiltration rate and average ultrafiltration rate by pre-dialysis weight.

All analyses were performed using R software (version 4.1.2; www.r-project.org; R Foundation for Statistical Computing, Vienna) and Python software (version 3.8; www.python.org; Python Software Foundation, Wilmington) with the PyTorch library. A computer with Xeon processor (24 core, Intel, Santa Clara, CA, USA) and Quadro RTX 6000 (Nvidia, Santa Clara, CA, USA) was used for all analyses.

## Results

### Characteristics of Hemodialysis Sessions

Of all hemodialysis sessions stored in the databases of two Korean hospitals (Severance Hospital and Myongji Hospital), 63,640 hemodialysis sessions were included (26,746 for internal validation, 36,894 for external validation) in our study. The baseline characteristics of the hemodialysis sessions are shown in Table 1. The mean dialysis time was 3.97 ± 0.18 h and 3.87 ± 0.30 h in Severance Hospital and Myongji Hospital, respectively. Total ultrafiltration was more likely lower in hemodialysis sessions performed at Severance Hospital than those performed at Myongji Hospital (2,287.36 ± 825.48 mL vs. 2,427.10 ± 880.45 mL). Pre-dialysis SBP was similar between the two hospitals (137.53 ± 21.84 mmHg vs. 137.89 ± 17.77 mmHg). Among 101,655 segments in the internal validation set, 3,755 (3.7%), 35,144 (34.6%), and 39,656 (39.0%) IDH events occurred according to three different IDH definitions (Nadir90, Fall20, and Fall20/MAP10, respectively). Similarly, 5,399 (2.2%), 99,844 (41.1%), and 110,018 (45.3%) IDH events occurred in the external validation set based on the Nadir90, Fall20, and Fall20/MAP10, respectively) (Table 2). The baseline characteristics of the patients are shown in Supplementary Table S2. In addition, we summarized the descriptive statistics of the variables according to the occurrence of IDH events (Supplementary Tables S3–S5).

**Table 1:** Characteristics of the study population.

**Variables**	**Severance hospital (*n =* 26,746)**	**Myongji hospital (*n =* 36,894)**
Total dialysis time, mean (SD), h	3.97 (0.18)	3.87 (0.30)
Arterial pressure, mean (SD), mmHg	−124.91 (29.66)	−139.11 (30.51)
Venous pressure, mean (SD), mmHg	118.25 (27.93)	123.34 (30.52)
Blood flow rate, mean (SD), mL/min	266.74 (30.86)	252.98 (25.17)
Average blood flow rate, mean (SD), mL/min	267.47 (30.92)	253.99 (24.56)
Dialysate flow rate, mean (SD), mL/min	573.90 (91.45)	552.64 (116.23)
Total ultrafiltration volume, mean (SD), mL	2,287.36 (825.48)	2,427.10 (880.45)
Ultrafiltration rate, mean (SD), mL/h	597.79 (215.32)	649.37 (228.27)
Average ultrafiltration rate, mean (SD), mL/h	597.94 (222.08)	653.12 (241.62)
Dialysate temperature, mean (SD), °C	36.05 (0.32)	36.21 (0.35)
Dialysate sodium level, mean (SD), mmol/L	139.20 (1.43)	140.35 (1.03)
Pre-dialytic SBP, mean (SD), mmHg	137.53 (21.84)	137.89 (17.77)
Pre-dialytic DBP, mean (SD), mmHg	64.61 (13.22)	65.80 (11.10)
Pre-dialytic MAP, mean (SD), mmHg	88.92 (13.79)	89.83 (11.27)
Pulse rate, mean (SD), beats per minute	69.91 (10.84)	71.62 (11.64)
Total number of segments	101,655	243,059

*SBP, systolic blood pressure; DBP, diastolic blood pressure; MAP, mean arterial pressure*.

**Table 2:** Number of events among segments according to the definitions of intradialytic hypotension.

**IDH**	**Severance hospital** **(*n =* 101,655)**	**Myongji hospital** **(*n =* 243,059)**
Nadir90, *n* (%)	3,755 (3.7)	5,399 (2.2)
Fall20, *n* (%)	35,144 (34.6)	99,844 (41.1)
Fall20/MAP10, *n* (%)	39,656 (39.0)	110,018 (45.3)

*IDH, intradialytic hypotension; MAP, mean arterial pressure; n, number of segments*.

### Model Performance

The model performance for internal and external validation is summarized in Table 3. First, we evaluated model performance for the dataset from Severance Hospital, using 5-fold cross-validation. In internal validation, our proposed deep learning model performed better than other models in terms of the AUROCs (Nadir90: 0.905, Fall20: 0.864, Fall20/MAP10: 0.863) and the AUPRC (Nadir90: 0.287, Fall20: 0.794, Fall20/MAP10: 0.812) in most cases. The logistic regression model showed the highest AUROC for Fall20 (0.868) and AUPRC for Nadir90 (0.298) for internal validation. Second, we conducted external validation using the Myongji Hospital dataset. In this validation, our proposed deep learning model showed the best performance by AUROC (Nadir90: 0.853, Fall20: 0.872, Fall20/MAP10: 0.853) and by AUPRC (Nadir90: 0.118, Fall20: 0.831, Fall20/MAP10: 0.841).

**Table 3:** Model performance for predicting intradialytic hypotension.

**IDH**	**Model**	**Internal validation**		**External validation**	
		**AUROC (min-max)**	**AUPRC (min-max)**	**AUROC (*p*-value)**	**AUPRC (*p*-value)**
Nadir90	DLM	0.905 (0.892–0.913)	0.287 (0.192–0.494)	0.853 (reference)	0.118 (reference)
	LR	0.900 (0.879–0.926)	0.298 (0.193–0.572)	0.833 (<0.001)	0.110 (0.056)
	RF	0.889 (0.847–0.903)	0.292 (0.192–0.566)	0.837 (<0.001)	0.115 (0.444)
	XGB	0.891 (0.855–0.906)	0.270 (0.140–0.582)	0.809 (<0.001)	0.089 (<0.001)
Fall20	DLM	0.864 (0.836–0.888)	0.794 (0.698–0.847)	0.872 (reference)	0.831 (reference)
	LR	0.868 (0.840–0.888)	0.788 (0.700–0.844)	0.855 (<0.001)	0.817 (<0.001)
	RF	0.844 (0.812–0.869)	0.750 (0.688–0.834)	0.850 (<0.001)	0.813 (<0.001)
	XGB	0.860 (0.820–0.873)	0.777 (0.701–0.812)	0.860 (<0.001)	0.815 (<0.001)
Fall20/MAP10	DLM	0.863 (0.827–0.878)	0.812 (0.729–0.858)	0.853 (reference)	0.841 (reference)
	LR	0.857 (0.825–0.873)	0.804 (0.726–0.854)	0.842 (<0.001)	0.827 (<0.001)
	RF	0.838 (0.801–0.859)	0.773 (0.720–0.827)	0.843 (<0.001)	0.827 (<0.001)
	XGB	0.851 (0.812–0.856)	0.795 (0.735–0.824)	0.843 (<0.001)	0.829 (<0.001)

*The performance measures of internal validation were calculated using 5-folds cross-validation; The min and max are the minimum and maximum values for 5 performance measures obtained through 5-folds cross-validation. P-values were calculated compared to the DLM. The Delong test was used to calculated p-values for comparison of AUROC. The bootstrap method was used to calculated p-values for comparison of AUPRC. IDH, intradialytic hypotension; MAP, mean arterial pressure; AUROC, area under the receiver operating characteristic curve; AUPRC, area under the precision-recall curve; LR, logistic regression; RF, random forest; XGB, extreme gradient boosting; DLM, deep learning model*.

### Variable Importance

We calculated overall variable importance using SHAP values for each definition of IDH to investigate which covariate most affected IDH (Supplementary Figures S1–S3). For all definitions of IDH, most vital signs were in the top ranks for variable importance. The blood flow rate, dialysate sodium, and AP were identified as the next most important variables, after SBP and MAP, for the Fall20 and Fall20/MAP10 IDH definitions. Also, the SHAP values can be used to determine whether variables decrease or increase the risks of IDH after 10 min in real time. The case-specific SHAP values over time were illustrated in Supplementary Figures S4–S6. The positive SHAP value was related to the decreased risk of IDH after 10 min, and vice versa if negative.

### Sensitivity Analyses

Sensitivity analyses were performed to determine which variables had more impact on IDH prediction for our deep learning model, using the external validation dataset (Table 4). The model including vital sign variables (SBP, DBP, MAP, and pulse rate) was defined as a reference model. For the Nadir90 definition, setting measure variables (blood flow rate, dialysate flow rate, ultrafiltration rate, total ultrafiltration volume, dialysate temperature, and dialysate sodium level) improved model performance more than other variable groups (AUROC: 0.819 to 0.839, AUPRC: 0.106 to 0.112). The variables of monitored pressure (AP, VP) increased model performance the most for the Fall20 (AUROC: 0.858 to 0.862, AUPRC: 0.823 to 0.829) and Fall20/MAP10 (AUROC: 0.848 to 0.853, AUPRC: 0.835 to 0.841) IDH definitions. Sensitivity analyses for the internal validation dataset showed similar results to those for the external validation dataset (Supplementary Table S6). In addition, demographic variables, including age and sex, did not have a significant influence on IDH prediction (Supplementary Table S7). There was no significant difference in the results according to the normalized method for ultrafiltration rate (Table 3 and Supplementary Table S8).

**Table 4:** Sensitivity analysis of deep learning model for external validation dataset.

**Variables**	**Nadir90**		**Fall20**		**Fall20/MAP10**	
	**AUROC (PC, *p*-value)**	**AUPRC (PC, *p*-value)**	**AUROC (PC, *p*-value)**	**AUPRC (PC, *p*-value)**	**AUROC (PC, *p*-value)**	**AUPRC (PC, *p*-value)**
Vital signs	0.819 (reference)	0.106 (reference)	0.858 (reference)	0.823 (reference)	0.848 (reference)	0.835 (reference)
Vital signs + Monitored pressure	0.835 (1.9, <0.001)	0.111 (5.1, 0.028)	0.862 (0.5, <0.001)	0.829 (0.7, 0.020)	0.853 (0.6, <0.001)	0.841 (0.7, 0.016)
Vital signs + Setting measures	0.839 (2.5, <0.001)	0.112 (6.1, 0.016)	0.858 (0.1, 0.081)	0.824 (0.1, 0.624)	0.849 (0.0, 0.227)	0.835 (0.0, 1.000)
Vital signs + Time setting	0.830 (1.3, <0.001)	0.108 (2.3, 0.566)	0.856 (−0.2, <0.001)	0.822 (−0.1, 0.636)	0.848 (-0.1, 0.012)	0.835 (0.0, 1.000)

*Vital signs included SBP, DBP, MAP, and pulse rate; Monitored pressure included AP, and VP; setting measures included blood flow rate, dialysate flow rate, ultrafiltration rate, total ultrafiltration volume, temperature, and dialysate sodium level. P-values were calculated compared to the models that were trained by only vital signs. The Delong test was used to calculated p-values for comparison of AUROC. The bootstrap method was used to calculated p-values for comparison of AUPRC. MAP, mean arterial pressure; AUROC, area under the receiver operating characteristic curves; AUPRC, area under the precision-recall curve; PC, percentage change*.

## Discussion

In this retrospective two-center study, we built a concise model to predict IDH 10 min ahead, using only data generated continuously during hemodialysis, without any risk of a breach of personal information. Because there was no significant difference in model performance improvement even in models with information on age and sex, our study suggests that the use of personal clinical data could be minimized when building a deep learning model, without significant loss of accuracy. In addition, no additional effort is needed to collect clinical data, other than the data extracted from the hemodialysis machine, for predicting IDH by this model. The variables used in this model are available from dialysis machines from any manufacturer. Furthermore, our model can predict IDH events as defined by three different definitions simultaneously and will be beneficial for individualized IDH prediction. Therefore, our model is cost-effective for predicting IDH in terms of privacy protection, individualized medicine, and generalizability.

IDH is associated with an increased risk of cardiovascular and all-cause mortality in end-stage kidney disease patients (1–4, 22). IDH is typically defined as a decrease in SBP ≥ 20 mmHg (23, 24) or a nadir in SBP < 90 mmHg (4). In a recent meta-analysis, the prevalence of IDH in hemodialysis sessions was about 10% (5). Known causes of IDH include excessive ultrafiltration, decreased cardiac output, and failure to increase vascular resistance. Several methods have been suggested to prevent or treat IDH, such as decreasing the ultrafiltration rate, avoiding marked interdialytic weight gain, increasing weekly treatment time, increasing the sodium concentration of the dialysate, lowering dialysate temperature, avoiding food intake during hemodialysis, using high-flux convection, and using midodrine (25). Patients with end-stage kidney disease have many risk factors for IDH that are difficult to correct. Therefore, it is more effective to prevent IDH early than to cope with IDH after IDH occurred. Moreover, particularly in patients with congestive heart failure, there is a risk a vicious cycle if interdialytic weight gain is not removed properly due to IDH. Consequently, early prediction or detection is important. However, as described earlier, it is difficult to predict IDH with the traditional statistical method, because the development of IDH is related to numerous factors, patients are diverse, and complex variables must be considered.

Recently, many studies developed models for predicting hypotension events during hemodialysis using machine learning or deep learning (6–8, 26). Prediction models developed in those studies require different types of clinical information to predict IDH. Depending on the hemodialysis center, these clinical data could be impossible to collect, or could be difficult to use due to privacy issues. Since there are no established guidelines about how the data collected are used and shared with others, protection of privacy is an important issue in the development and use of machine learning or deep learning algorithms (27). In this respect, our model differs from other prediction models, because it does not use any personal information other than the monitoring data obtained from the hemodialysis machine. In addition, our model has the advantage that it needs a minimal number of variables, without accessing electronic medical records, and there is no additional cost for data merging from different platforms, allowing it to be applied in hemodialysis centers of various sizes. Furthermore, our model was designed to predict IDH after 10 min, so that medical staff in the hemodialysis center can effectively conduct acute management, including use of the Trendelenburg position, isotonic fluid administration, and reducing ultrafiltration rate, in advance. Although deep learning models need more computing power than other models including linear regression, XGBoost, and random forest, researchers might expect to monitor in real-time how variables affect IDH in deep learning models through SHAP value. It would be necessary to conduct further studies in order to investigate that our models could reduce the risk of IDH in real world. Moreover, real-time data at 1-min intervals were used to detect IDH events in this study. In some studies, time-varying variables were used to improve the efficacy of the prediction model, but real-time data continuously generated from the dialysis machine were not used, as in our study (8). Similarly, a previous study showed the feasibility of using data continuously measured by the hemodialysis machine to predict outcomes (28).

There were some limitations to our study. First, this study was a small retrospective study, using a hemodialysis database. Although it was a multicenter study, the number of participants was relatively small. Second, our model may not be suitable for predicting IDH in inpatients, since our study was conducted based on outpatient clinic data. In this reason, the number of blood pressure measurements is inevitably limited because unnecessary measurements of blood pressure could make the patients feel uncomfortable during hemodialysis. More frequent blood pressure measurements would be needed to build a more accurate model especially for inpatients. However, most of the patients with chronic hemodialysis undergo hemodialysis at outpatient clinics and preventing adverse events in these patients is more cost-effective. Third, due to the nature of the database platform used in this study, our model was based on data generated at 1-min intervals, rather than in real-time. In addition, a hardware interface that collects data from a hemodialysis machine is needed to implement our model. However, our model does not use any sensitive personal information, it may not be difficult to develop a platform for data collection and utilization. Fourth, the real-time monitoring data collected from hemodialysis machine in this study was anonymized and could not be merged with electronic medical record. Only limited clinical information including gender, and age can be merged. Although our models showed relatively good performance, the comparison with the models including clinical information was not possible in this study in this reason. Fifth, IDH event defined as decrease in blood pressure associated with symptoms and/or interventions were not considered due to the limitation of database. Further well-designed studies would be needed to consider symptoms associated with hypotension event. Finally, some of our models had low AUPRC as compared with other models. The frequency of Nadir90 events were reported as 9.7–11.3% among hemodialysis sessions in another large hemodialysis cohort (22). In contrast, only 2.2–3.7% of Nadir90 events were observed in our study. The relatively low AUPRC value for a certain outcome is thought to be due to the low incidence of these events. Despite the low AUPRC in one outcome, it might be helpful for clinicians to apply an individualized treatment, because our model allows prediction of IDH by various definitions simultaneously.

In conclusion, our study showed that IDH is sufficiently and accurately predictable using a deep learning model, without including any sensitive personal information. In addition, the variables used in our model can be obtained at any hemodialysis center globally, making it more generalizable than existing models. We expect that our model can be prospectively validated and used in various situations for the safety of hemodialysis patients.

## Data Availability Statement

The datasets presented in this article are not readily available because the data underlying this article cannot be shared to other academics due to the privacy of individuals that participated in the study. Requests to access the datasets should be directed to HWK, drhwint@yuhs.ac.

## Ethics Statement

The studies involving human participants were reviewed and approved by the Institutional Review Boards of Severance Hospital and Myongji Hospital. The Ethics Committee waived the requirement of written informed consent for participation.

## Author Contributions

HWK and BSK contributed to the research concept and the study design. HWK, BSK, SIB, YEK, HMC, and D-JO were involved in data acquisition and cleansing. HWK, S-JH, MK, JL, KHP, and GL contributed to the statistical analyses and model building. HWK and S-JH wrote the manuscript. BSK and C-MN were responsible for data analysis and interpretation and supervision or mentorship. All authors read and approved the final version of the manuscript.

## Funding

This study was supported by a Severance Hospital Research fund for Clinical excellence (SHRC) (C-2020-0011).

## Conflict of Interest

The authors declare that the research was conducted in the absence of any commercial or financial relationships that could be construed as a potential conflict of interest.

## Publisher's Note

All claims expressed in this article are solely those of the authors and do not necessarily represent those of their affiliated organizations, or those of the publisher, the editors and the reviewers. Any product that may be evaluated in this article, or claim that may be made by its manufacturer, is not guaranteed or endorsed by the publisher.
