# Supplementary material for: Deep Learning Model for Predicting Intradialytic Hypotension Without Privacy Infringement: A Retrospective Two-Center Study
Source: Front Med (Lausanne). 2022 Jul 7;9:878858. doi: 10.3389/fmed.2022.878858 (PMC9300869; doi:10.3389/fmed.2022.878858)
Supplement: Supplementary file 1 [file Data_Sheet_1.docx]

Supplementary Material

**Deep learning model for predicting intradialytic hypotension regardless of privacy infringement: a retrospective two center study**

Hyung Woo Kim, MD^1, †^, Seok-Jae Heo, MS^2, †^, Minseok Kim^2^, Jakyung Lee^2^, Keun Hyung Park, MD^1^, Gongmyung Lee, MD^1^, Song In Baeg, MD^3^, Young Eun Kwon, MD, PhD^3^, Hye Min Choi, MD, PhD^3^, Dong-Jin Oh, MD, PhD^3^, Chung-Mo Nam, PhD^2,4^, and Beom Seok Kim, MD, PhD^1^

*^1^Department of Internal Medicine, Yonsei University College of Medicine, Seoul, Korea*

*^2^Department of Biostatistics and Computing, Yonsei University Graduate School, Seoul, Korea*

*^3^Department of Internal Medicine, Hanyang University College of Medicine, Myongji Hospital, Goyang, Korea*

*^4^Division of Biostatistics, Department of Biomedical Systems Informatics, Yonsei University College of Medicine, Seoul, Korea*

**†These authors have contributed equally to this work.**

*** Correspondence:**Beom Seok Kim MD, PhD (Main corresponding)

[docbsk@yuhs.ac](mailto:docbsk@yuhs.ac)

Chung-Mo Nam PhD

cmnam@yuhs.ac

***Table S1.*** Summary of hyperparameters for random forest and XGBoost.

| **Model** | **Hyperparameter** | **Description** | **Value** |
| --- | --- | --- | --- |
| RF | n_estimators | The number of trees in the forest | 100 |
|  | criterion | The function to measure the quality of a split | Gini |
|  | max_depth | The maximum depth of the tree. If None, then nodes are expanded untill all leaves are pure or until all leaves contain less than min_samples_split samples | None |
|  | min_samples_split | The minimum number of samples required to split an internal node | 2 |
|  | min_samples_leaf | The minimum number of samples required to be at a leaf node | 1 |
| XGBoost | n_estimators | Number of boosting rounds | 100 |
|  | max_depth | Maximum tree depth for base learners | 6 |
|  | booster | Type of booster | gbtree |
|  | learning_rate | Step size shirinkage | 0.3 |
|  | colsample_bytree | Subsample ratio of columns when constructing each tree | 1 |
|  | colsample_bylevel | Subsample ratio of columns for each level | 1 |
|  | colsample_bynode | Subsample ratio of columns for each node | 1 |
|  | reg_alpha | L1 regularization term of weights | 0 |
|  | reg_lambda | L2 regularization term of weights | 1 |
|  | subsample | Subsample ratio of the training instances | 1 |
|  | gamma | Minimum loss reduction required to make a futher partition on a leaf node of the tree | 0 |
| ***Abbreviations***: RF, random forest; XGB, extreme gradient boosting. | | | |

***Table S2.*** Descriptive statistics of demographic variables for age and gender.

| **Variables** | **Severance Hospital (n=79)** | **Myongji Hospital (n=255)** | **p-value** |
| --- | --- | --- | --- |
| Age, mean (SD) | 62.2 (16.7) | 67.7 (12.7) | 0.008 |
| Gender, n (%) |  |  | 0.237 |
| Male | 38 (48.1%) | 144 (56.5%) |  |
| Female | 41 (51.9%) | 111 (43.5%) |  |
| ***Note***: Student's t-test and Pearson's chi-squared test are used to calculate p-values. | | | |

***Table S3.*** Descriptive statistics of hemodialysis-related measurements according to occurrence of Nadir90 for internal validation dataset.

| **Variables** | **Nadir90** | | |
| --- | --- | --- | --- |
|  | **Yes (n=3,755)** | **No (n=97,900)** | **p-value** |
| Change of arterial pressure, mmHg | 0.37 (10.48) | -0.50 (8.42) | <0.001 |
| Change of venous pressure, mmHg | -1.55 (9.50) | 0.04 (9.47) | <0.001 |
| Change of blood flow rate, mL/min | -3.17 (10.79) | -0.57 (5.40) | <0.001 |
| Change of average blood flow rate, mL/min | -0.75 (2.00) | 0.04 (1.39) | <0.001 |
| Change of dialysate flow rate, mL/min | -3.86 (31.15) | -2.10 (31.04) | <0.001 |
| Change of total ultrafiltration volume, mL | 155.81 (50.04) | 133.87 (50.74) | <0.001 |
| Change of ultrafiltration rate, mL/h | -19.55 (62.72) | -8.10 (42.70) | <0.001 |
| Change of average ultrafiltration rate, mL/h | -6.86 (12.20) | -2.25 (8.01) | <0.001 |
| Change of dialysate temperature, ℃ | -0.01 (0.20) | 0.00 (0.21) | 0.122 |
| Change of dialysate sodium level, mmol/L | 0.04 (0.26) | 0.01 (0.17) | <0.001 |
| Pre-dialytic SBP, mmHg | 121.76 (25.24) | 147.40 (25.29) | <0.001 |
| Pre-dialytic DBP, mmHg | 55.80 (13.28) | 64.21 (15.92) | <0.001 |
| Pre-dialytic MAP, mmHg | 77.79 (14.76) | 91.94 (16.19) | <0.001 |
| Pulse rate, beats per minute | 76.97 (11.27) | 70.54 (11.59) | <0.001 |
| ***Note***: The change value is the average value of the segment unit calculated based on the initial value of the segment; The descriptive statistics are expressed as mean (SD); Student's t-test is used to calculate p-values.  ***Abbreviations***: MAP, mean arterial pressure. | | | |

***Table S4.*** Descriptive statistics of hemodialysis-related measurements according to occurrence of Fall20 for internal validation dataset.

| **Variables** | **Fall20** | | |
| --- | --- | --- | --- |
|  | **Yes (n=35,144)** | **No (n=66,511)** | **p-value** |
| Change of arterial pressure, mmHg | -0.45 (8.82) | -0.48 (8.34) | 0.626 |
| Change of venous pressure, mmHg | -0.51 (9.41) | 0.24 (9.51) | <0.001 |
| Change of blood flow rate, mL/min | -1.17 (6.91) | -0.39 (4.94) | <0.001 |
| Change of average blood flow rate, mL/min | -0.15 (1.45) | 0.09 (1.40) | <0.001 |
| Change of dialysate flow rate, mL/min | -2.36 (30.70) | -2.06 (31.22) | 0.144 |
| Change of total ultrafiltration volume, mL | 145.66 (49.65) | 128.88 (50.57) | <0.001 |
| Change of ultrafiltration rate, mL/h | -11.59 (52.30) | -6.91 (38.20) | <0.001 |
| Change of average ultrafiltration rate, mL/h | -3.88 (8.96) | -1.66 (7.74) | <0.001 |
| Change of dialysate temperature, ℃ | 0.00 (0.21) | 0.00 (0.21) | 0.050 |
| Change of dialysate sodium level, mmol/L | 0.02 (0.23) | 0.00 (0.14) | <0.001 |
| Pre-dialytic SBP, mmHg | 160.59 (25.77) | 138.98 (22.37) | <0.001 |
| Pre-dialytic DBP, mmHg | 66.74 (16.74) | 62.40 (15.25) | <0.001 |
| Pre-dialytic MAP, mmHg | 98.02 (16.75) | 87.93 (15.01) | <0.001 |
| Pulse rate, beats per minute | 73.28 (11.79) | 69.46 (11.35) | <0.001 |
| ***Note***: The change value is the average value of the segment unit calculated based on the initial value of the segment; The descriptive statistics are expressed as mean (SD); Student's t-test is used to calculate p-values.  ***Abbreviations***: MAP, mean arterial pressure. | | | |

***Table S5.*** Descriptive statistics of hemodialysis-related measurements according to occurrence of Fall20/MAP10 for internal validation dataset.

| **Variables** | **Fall20/MAP10** | | |
| --- | --- | --- | --- |
|  | **Yes (n=39,656)** | **No (n=61,999)** | **p-value** |
| Change of arterial pressure, mmHg | -0.46 (8.73) | -0.47 (8.37) | 0.816 |
| Change of venous pressure, mmHg | -0.50 (9.38) | 0.29 (9.53) | <0.001 |
| Change of blood flow rate, mL/min | -1.13 (6.81) | -0.37 (4.85) | <0.001 |
| Change of average blood flow rate, mL/min | -0.13 (1.44) | 0.10 (1.41) | <0.001 |
| Change of dialysate flow rate, mL/min | -2.33 (30.69) | -2.06 (31.27) | 0.180 |
| Change of total ultrafiltration volume, mL | 144.55 (50.42) | 128.37 (50.18) | <0.001 |
| Change of ultrafiltration rate, mL/h | -11.39 (51.45) | -6.70 (37.72) | <0.001 |
| Change of average ultrafiltration rate, mL/h | -3.75 (8.85) | -1.58 (7.72) | <0.001 |
| Change of dialysate temperature, ℃ | 0.00 (0.21) | 0.00 (0.21) | 0.010 |
| Change of dialysate sodium level, mmol/L | 0.02 (0.23) | 0.00 (0.13) | <0.001 |
| Pre-dialytic SBP, mmHg | 158.53 (26.16) | 138.72 (22.27) | <0.001 |
| Pre-dialytic DBP, mmHg | 67.59 (16.93) | 61.54 (14.75) | <0.001 |
| Pre-dialytic MAP, mmHg | 97.91 (16.69) | 87.27 (14.70) | <0.001 |
| Pulse rate, beats per minute | 73.30 (11.99) | 69.17 (11.12) | <0.001 |
| ***Note***: The change value is the average value of the segment unit calculated based on the initial value of the segment; The descriptive statistics are expressed as mean (SD); Student's t-test is used to calculate p-values.  ***Abbreviations***: MAP, mean arterial pressure. | | | |

***Table S6.*** Sensitivity analysis of deep learning model for internal validation dataset.

| **Variables** | **Nadir90** | | **Fall20** | | **Fall20/MAP10** | |
| --- | --- | --- | --- | --- | --- | --- |
|  | AUROC  (PC, p-value) | AUPRC  (PC, p-value) | AUROC  (PC, p-value) | AUPRC  (PC, p-value) | AUROC  (PC, p-value) | AUPRC  (PC, p-value) |
| Vital signs | 0.890  (reference) | 0.302  (reference) | 0.863  (reference) | 0.785  (reference) | 0.855  (reference) | 0.803  (reference) |
| Vital signs + Monitored pressure | 0.894  (0.5, 0.026) | 0.302  (-0.1, 0.873) | 0.869  (0.8, <0.001) | 0.791  (0.8, <0.001) | 0.861  (0.7, <0.001) | 0.809  (0.7, 0.012) |
| Vital signs + Setting measures | 0.893  (0.4, 0.035) | 0.306  (1.4, <0.001) | 0.862  (-0.1, 0.393) | 0.780  (-0.6, 0.049) | 0.853  (-0.2, 0.192) | 0.799  (-0.5, 0.052) |
| Vital signs + Time setting | 0.900  (1.1, 0.008) | 0.311  (2.9, <0.001) | 0.866  (0.4, 0.119) | 0.787  (0.3, 0.440) | 0.859  (0.5, 0.027) | 0.806  (0.4, 0.234) |
| ***Note:*** Vital signs included systolic blood pressure, diastolic blood pressure, mean arterial pressure, and pulse rate; Monitored pressure included atrial pressure and venous pressure; setting measures included blood flow rate, dialysate flow rate, ultrafiltration rate, total ultrafiltration volume, temperature, and dialysate sodium level. P-values were calculated compared to the models that were trained by only vital signs. The Delong test was used to calculated p-values for comparison of AUROC. The bootstrap method was used to calculated p-values for comparison of AUPRC.  ***Abbreviations***: MAP, mean arterial pressure; AUROC, area under the receiver operating characteristic curve; AUPRC, area under the precision-recall curve; PC, percentage change. | | | | | | |

***Table S7.*** Model performance for predicting intradialytic hypotension including information of age and gender.

| **IDH** | **Model** | **Internal validation** | | **External validation** | |
| --- | --- | --- | --- | --- | --- |
|  |  | AUROC (min-max) | AUPRC (min-max) | AUROC (p-value) | AUPRC (p-value) |
| Nadir90 | DLM | 0.898 (0.881-0.913) | 0.295 (0.089-0.635) | 0.848 (reference) | 0.109 (reference) |
|  | LR | 0.895 (0.872-0.924) | 0.275 (0.097-0.622) | 0.825 (<0.001) | 0.102 (0.042) |
|  | RF | 0.871 (0.846-0.901) | 0.239 (0.064-0.581) | 0.830 (<0.001) | 0.112 (0.498) |
|  | XGB | 0.882 (0.855-0.906) | 0.236 (0.067-0.582) | 0.813 (<0.001) | 0.089 (<0.001) |
| Fall20 | DLM | 0.872 (0.842-0.887) | 0.794 (0.729-0.833) | 0.866 (reference) | 0.832 (reference) |
|  | LR | 0.858 (0.837-0.877) | 0.775 (0.734-0.820) | 0.855 (<0.001) | 0.817 (<0.001) |
|  | RF | 0.827 (0.811-0.869) | 0.717 (0.684-0.792) | 0.842 (<0.001) | 0.803 (<0.001) |
|  | XGB | 0.846 (0.820-0.873) | 0.750 (0.701-0.812) | 0.847 (<0.001) | 0.807 (<0.001) |
| Fall20/MAP10 | DLM | 0.862 (0.834-0.871) | 0.810 (0.774-0.831) | 0.854 (reference) | 0.841 (reference) |
|  | LR | 0.846 (0.826-0.861) | 0.792 (0.755-0.825) | 0.841 (<0.001) | 0.827 (<0.001) |
|  | RF | 0.823 (0.792-0.859) | 0.748 (0.714-0.811) | 0.833 (<0.001) | 0.817 (<0.001) |
|  | XGB | 0.835 (0.812-0.860) | 0.771 (0.735-0.824) | 0.831 (<0.001) | 0.817 (<0.001) |
| ***Abbreviations***: IDH, intradialytic hypotension; MAP, mean arterial pressure; AUROC, area under the receiver operating characteristic curve; AUPRC, area under the precision-recall curve; LR, logistic regression; RF, random forest; XGB, extreme gradient boosting; DLM, deep learning model.  ***Notes***: The performance measures of internal validation were calculated using five-fold cross-validation; The min and max are the minimum and maximum values for 5 performance measures obtained through 5-folds cross-validation. P-values were calculated compared to the DLM. The Delong test was used to calculated p-values for comparison of AUROC. The bootstrap method was used to calculated p-values for comparison of AUPRC. | | | | | |

***Table S8.*** Model performance for predicting intradialytic hypotension using normalized ultrafiltration rate and average ultrafiltration rate by pre-dialysis weight.

| **IDH** | **Model** | **Internal validation** | | **External validation** | |
| --- | --- | --- | --- | --- | --- |
|  |  | AUROC (min-max) | AUPRC (min-max) | AUROC (p-value) | AUPRC (p-value) |
| Nadir90 | DLM | 0.904 (0.884-0.929) | 0.308 (0.122-0.546) | 0.836 (reference) | 0.110 (reference) |
|  | LR | 0.895 (0.823-0.930) | 0.324 (0.108-0.586) | 0.829 (<0.001) | 0.106 (0.113) |
|  | RF | 0.890 (0.850-0.919) | 0.298 (0.101-0.577) | 0.829 (<0.001) | 0.101 (0.016) |
|  | XGB | 0.883 (0.853-0.928) | 0.262 (0.086-0.463) | 0.832 (0.042) | 0.107 (0.472) |
| Fall20 | DLM | 0.860 (0.838-0.876) | 0.777 (0.714-0.804) | 0.857 (reference) | 0.816 (reference) |
|  | LR | 0.856 (0.840-0.872) | 0.773 (0.697-0.807) | 0.850 (<0.001) | 0.821 (0.064) |
|  | RF | 0.831 (0.804-0.855) | 0.725 (0.623-0.761) | 0.845 (<0.001) | 0.804 (<0.001) |
|  | XGB | 0.846 (0.808-0.871) | 0.757 (0.680-0.783) | 0.849 (<0.001) | 0.803 (<0.001) |
| Fall20/MAP10 | DLM | 0.851 (0.832-0.864) | 0.798 (0.744-0.824) | 0.854 (reference) | 0.842 (reference) |
|  | LR | 0.844 (0.833-0.855) | 0.791 (0.726-0.830) | 0.847 (0.006) | 0.833 (0.011) |
|  | RF | 0.823 (0.799-0.845) | 0.752 (0.670-0.786) | 0.836 (<0.001) | 0.819 (<0.001) |
|  | XGB | 0.836 (0.805-0.847) | 0.777 (0.716-0.803) | 0.849 (0.038) | 0.835 (0.043) |
| ***Abbreviations***: IDH, intradialytic hypotension; MAP, mean arterial pressure; AUROC, area under the receiver operating characteristic curve; AUPRC, area under the precision-recall curve; LR, logistic regression; RF, random forest; XGB, extreme gradient boosting; DLM, deep learning model.  ***Notes***: The performance measures of internal validation were calculated using 5-folds cross-validation; The min and max are the minimum and maximum values for 5 performance measures obtained through 5-folds cross-validation. P-values were calculated compared to the DLM. The Delong test was used to calculated p-values for comparison of AUROC. The bootstrap method was used to calculated p-values for comparison of AUPRC. | | | | | |

***Figure S1.*** Variable importance obtained by SHAP values for Nadir90.


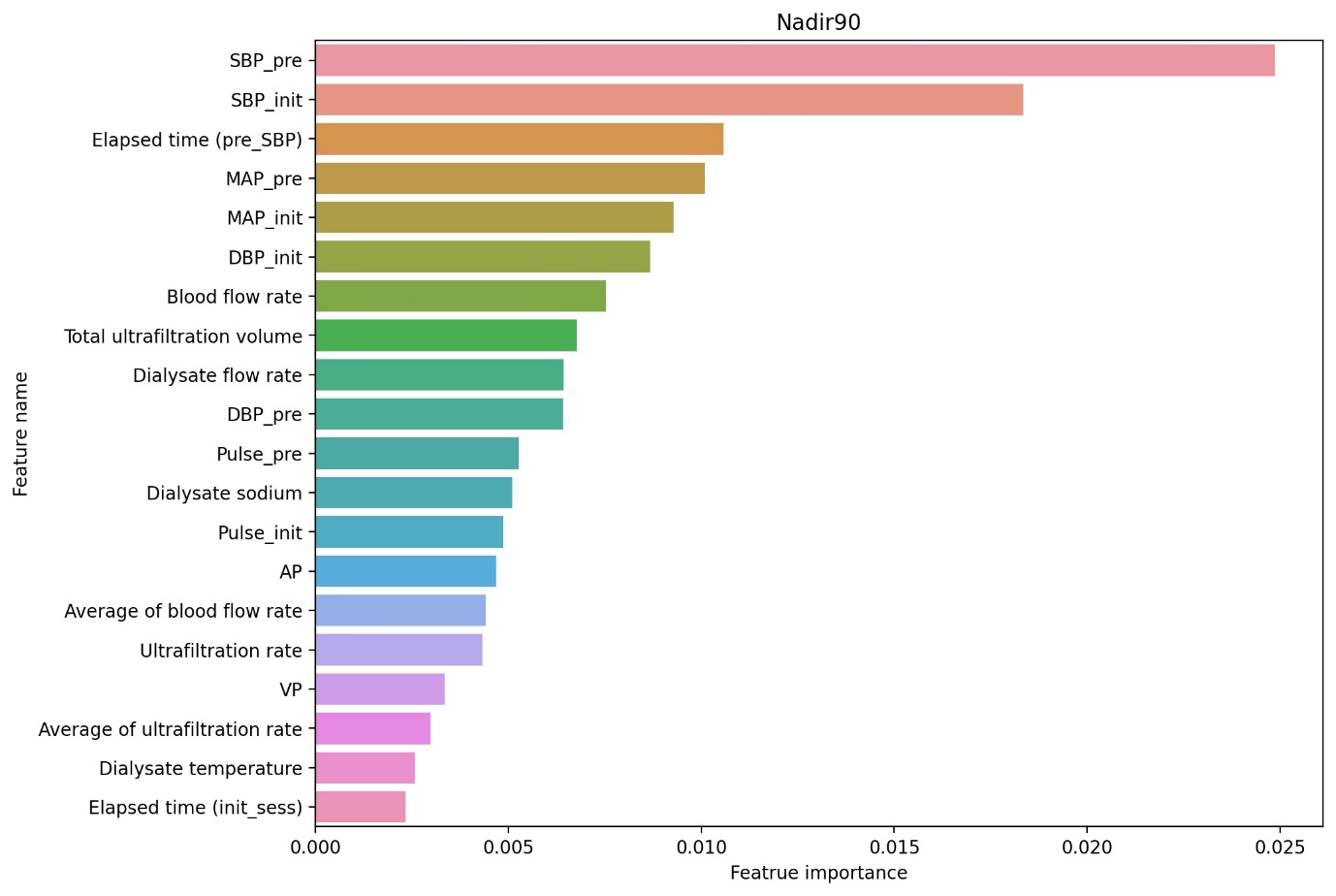


***Abbreviations:*** SHAP, SHapley Additive exPlanation; SBP, systolic blood pressure; SBP_pre, SBP measurement of previous segment; SBP_init, initial SBP measurement in each session; DBP, diastolic blood pressure; DBP_pre, DBP measurement of previous segment; DBP_init, initial DBP measurement in each session; MAP, mean arterial pressure; MAP_pre, MAP measurement of previous segment; MAP_init, initial MAP measurement in each session; Pulse_pre, pulse rate measurement of previous segment; Pulse_init, initial pulse rate measurement in each session; AP, arterial pressure; VP, venous pressure.

***Notes:*** Variable importance was calculated by the sum of absolute SHAP values over time for each variable.

***Figure S2.*** Variable importance obtained by SHAP values for Fall20.


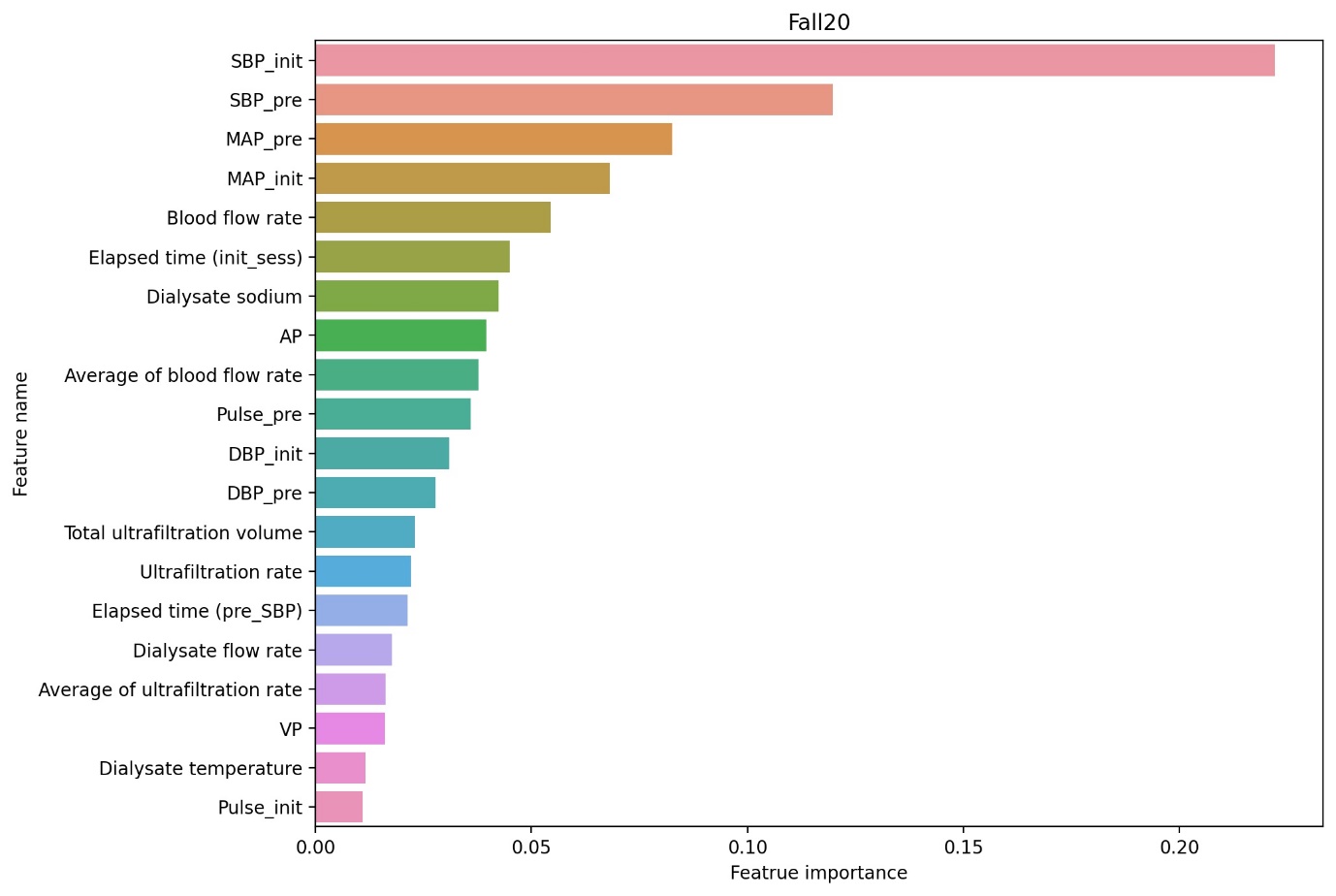


***Abbreviations:*** SHAP, SHapley Additive explanation; SBP, systolic blood pressure; SBP_pre, SBP measurement of previous segment; SBP_init, initial SBP measurement in each session; DBP, diastolic blood pressure; DBP_pre, DBP measurement of previous segment; DBP_init, initial DBP measurement in each session; MAP, mean arterial pressure; MAP_pre, MAP measurement of previous segment; MAP_init, initial MAP measurement in each session; Pulse_pre, pulse rate measurement of previous segment; Pulse_init, initial pulse rate measurement in each session; AP, arterial pressure; VP, venous pressure.

***Notes:*** Variable importance was calculated by the sum of absolute SHAP values over time for each variable.

***Figure S3.*** Variable importance obtained by SHAP values for Fall20/MAP10.


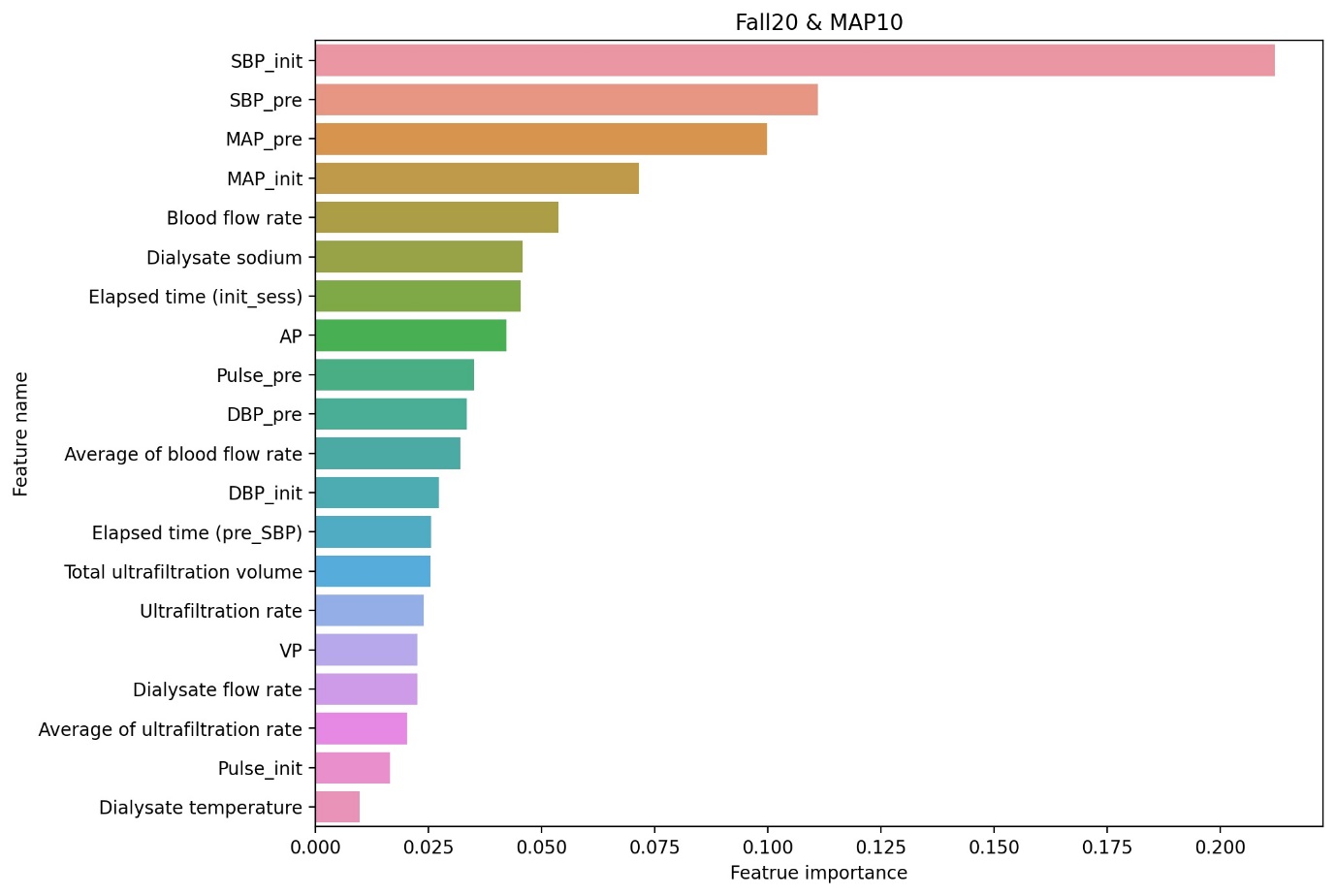


***Abbreviations:*** SHAP, SHapley Additive explanation; SBP, systolic blood pressure; SBP_pre, SBP measurement of previous segment; SBP_init, initial SBP measurement in each session; DBP, diastolic blood pressure; DBP_pre, DBP measurement of previous segment; DBP_init, initial DBP measurement in each session; MAP, mean arterial pressure; MAP_pre, MAP measurement of previous segment; MAP_init, initial MAP measurement in each session; Pulse_pre, pulse rate measurement of previous segment; Pulse_init, initial pulse rate measurement in each session; AP, arterial pressure; VP, venous pressure.

***Notes:*** Variable importance was calculated by the sum of absolute SHAP values over time for each variable.

***Figure S4.*** Individualized prediction with interpretation for Nadir90.

**
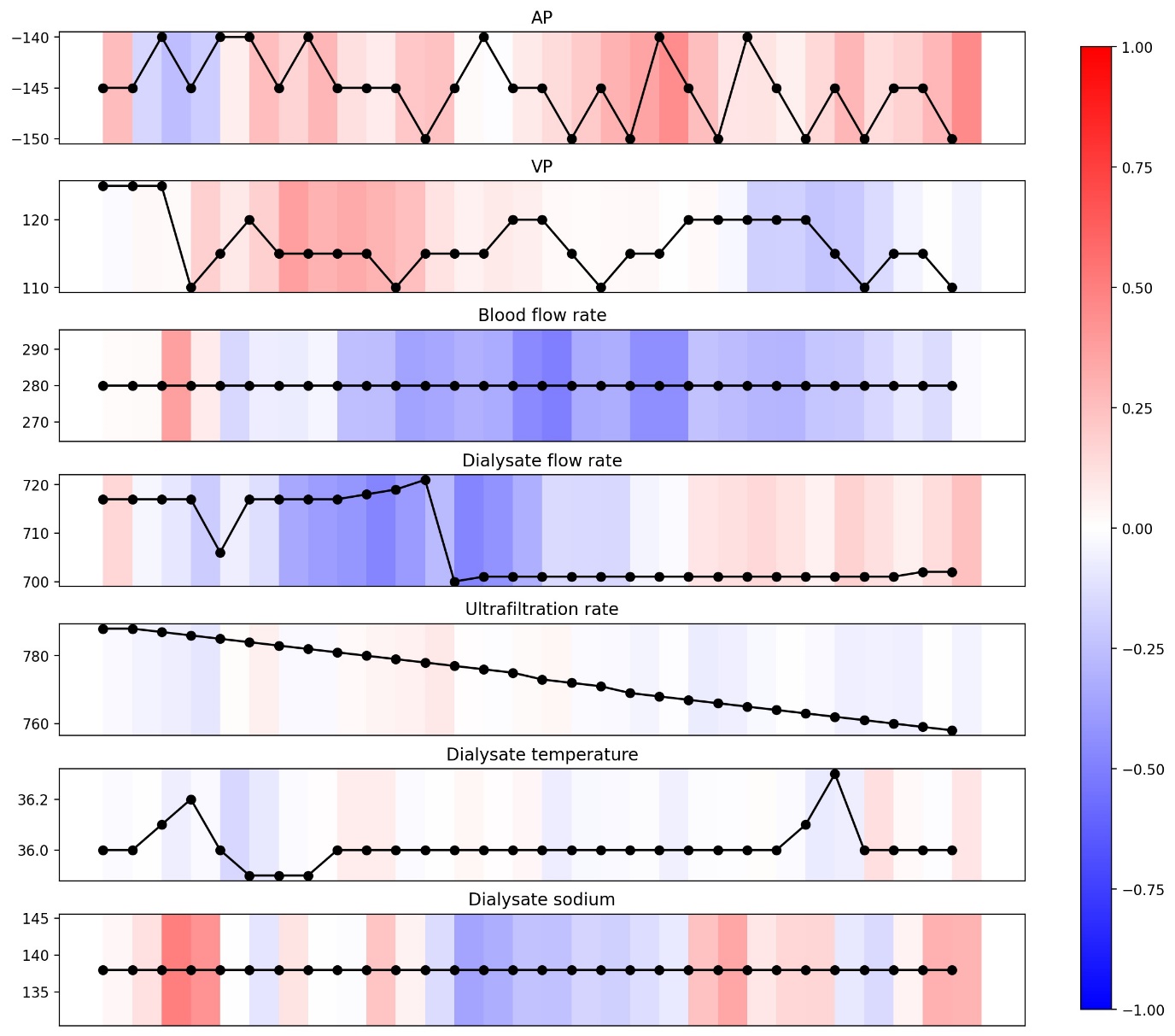
**

***Abbreviations:*** AP, arterial pressure; VP, venous pressure.

***Notes:*** SHAP values scaled from -1 to 1 were used a relative risk corresponding to gradient color for Nadir90. The red color indicates that the variable value at a specific time point increases the risk of Nadir90. The blue color indicates that the variable value at a specific time point decreases the risk of Nadir90.

***Figure S5.*** Individualized prediction with interpretation for Fall20.


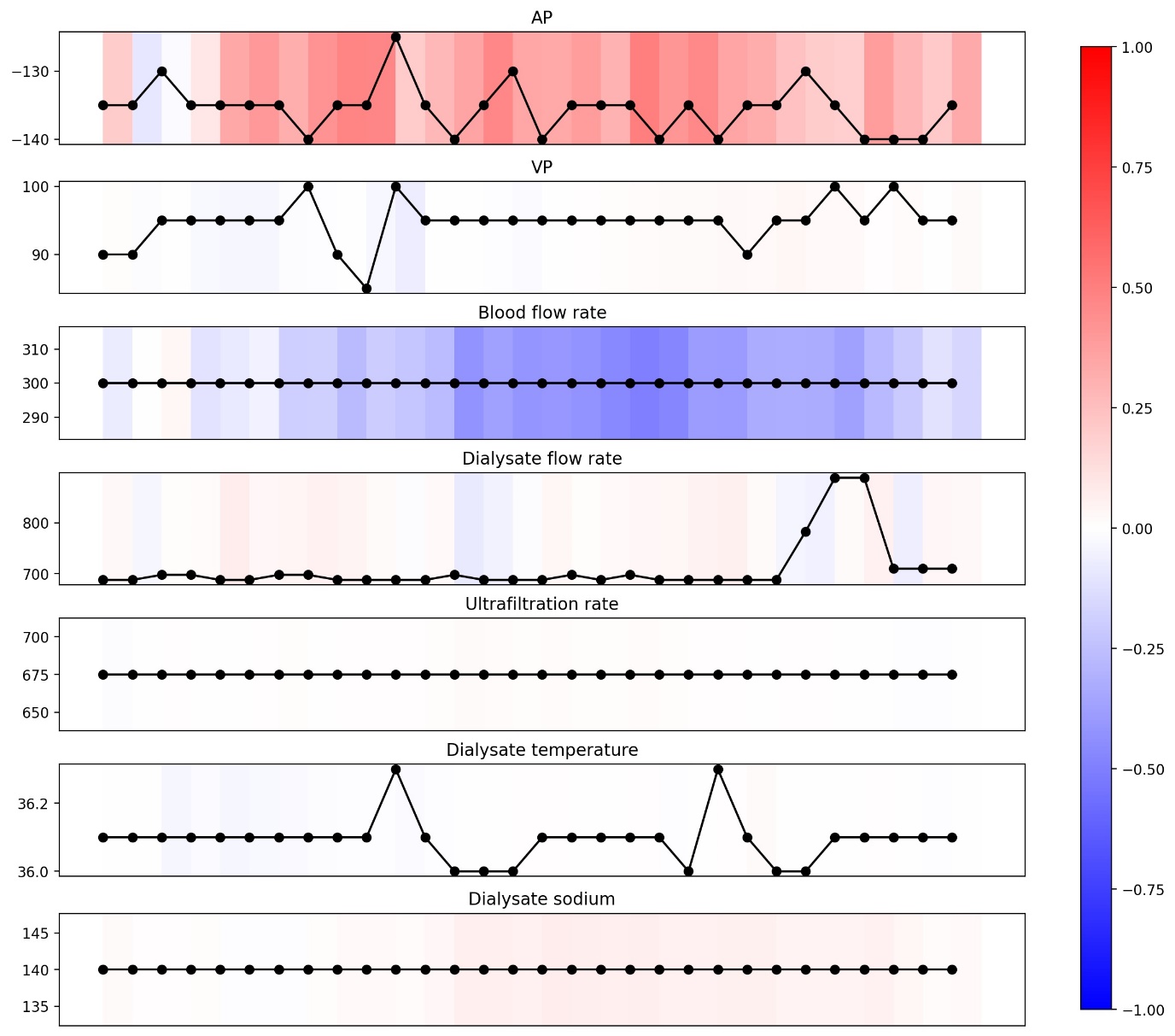


***Abbreviations:*** AP, arterial pressure; VP, venous pressure.

***Notes:*** SHAP values scaled from -1 to 1 were used a relative risk corresponding to gradient color for Fall20. The red color indicates that the variable value at a specific time point increases the risk of Fall20. The blue color indicates that the variable value at a specific time point decreases the risk of Fall20.

***Figure S6.*** Individualized prediction with interpretation for Fall20/MAP10.


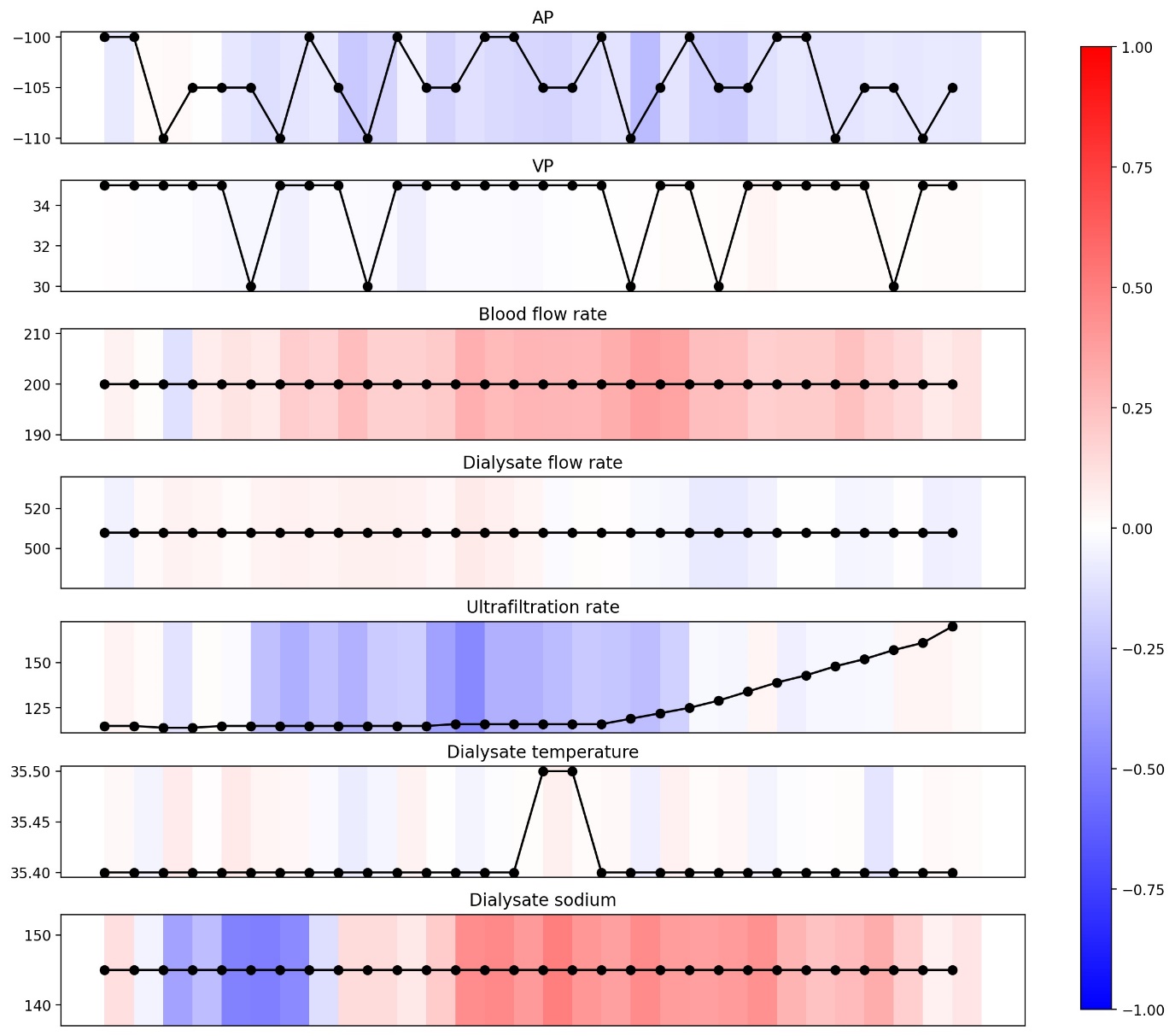


***Abbreviations:*** AP, arterial pressure; VP, venous pressure.

***Notes:*** SHAP values scaled from -1 to 1 were used a relative risk corresponding to gradient color for Fall20 or MAP10. The red color indicates that the variable value at a specific time point increases the risk of Fall20 or MAP10. The blue color indicates that the variable value at a specific time point decreases the risk of Fall20 or MAP10.
